# Optimizing Contraceptive Prescription in Smoking Women: A Quality Improvement Study

**DOI:** 10.7759/cureus.45701

**Published:** 2023-09-21

**Authors:** Catarina Novais, Joaquim Santos, Claúdia Alves, Ema Mendonça, João Salgado, Diogo Lopes, Ana Margarida Cruz

**Affiliations:** 1 Family Medicine, USF Bom Porto - ACeS Porto Ocidental, Porto, PRT

**Keywords:** combined hormonal contraceptives, smoking, women’s health, quality improvement, medical eligibility criteria, medication safety, primary care

## Abstract

Introduction

Family planning is fundamental in primary care (PC), and informed counseling helps to guide contraceptive choices. Combined hormonal contraceptives (CHC) pose cardiovascular risks, particularly when combined with smoking. According to the World Health Organization, the recommended global clinical decision is to refrain from employing CHC in female smokers aged 35 or older. This study aimed to improve contraception prescription for female smokers aged 35-54 in PC.

Methods

This study was conducted in a Portuguese Family Health Unit (FHU) from October 2021 to August 2022, and it followed the Standards for Quality Improvement Reporting Excellence (SQUIRE 2.0) guidelines and the Plan-Do-Study-Act approach. Female smokers aged 35-54 were included and evaluated in three moments: baseline evaluation (October 2021), intermediate evaluation (February 2022), and post-intervention evaluation (August 2022). Interventions involved educating medical staff, creating patient awareness campaigns, and evaluating contraceptive methods. The established quality-defining goal was to decrease the prevalence of female smokers aged 35 or above using CHC to ≤5%.

Results

CHC prevalence among smoking women aged 35-54 decreased from 16.4% to 8.5% after nine months of the initial intervention. There was an increase in progestogen-only methods usage over time, from 25.7% to 34.0%.

Discussion

Despite not fully achieving the predefined goal, interventions led to a substantial reduction of CHC use among smoking women aged 35-54. Collaborative efforts between healthcare professionals and patients played a pivotal role in optimizing care and reducing cardiovascular risk. This study underscores the importance of continuous quality improvement, collaborative interventions, and knowledge updates in Family Medicine practice. While conducted in a single FHU, the intervention’s multidimensional approach holds the potential for broader applicability.

Conclusion

In the future, healthcare teams should continue to reflect on achieved results, maintain knowledge, and empower patients in contraceptive method choices. The study contributes to improved care quality and highlights the positive impact on medical practice and patient outcomes.

## Introduction

In primary care (PC), family planning constitutes a fundamental component of a family doctor’s clinical practice, with counseling being one of its pillars. Through the clarification of risks and benefits associated with each contraceptive method, informed choice is facilitated, allowing the selection of the most suitable method based on each individual’s characteristics [1]. This approach facilitates adherence and continuity of the use of the chosen method. Therefore, it is imperative that family healthcare teams, including doctors and nurses, acquire up-to-date knowledge regarding the eligibility criteria and contraindications for each contraceptive method.

Worldwide, combined hormonal contraceptives (CHC) are a frequently employed contraceptive method among women of reproductive age, involving the combination of estrogen and progestin [2]. These methods are available in three different formulations: oral, transdermal patch, and vaginal ring, all of which have the same eligibility criteria according to the World Health Organization (WHO) [3].

Based on the most recent evidence, the use of CHC is associated with a higher risk of not only venous thromboembolism but also myocardial infarction (MI) [3]. These contraceptive methods in combination with smoking significantly increase the risk of MI, particularly after the age of 35, being directly correlated with the number of cigarettes smoked per day [4-7]. These findings are of extraordinary importance considering that, in 2019, the prevalence of daily tobacco use among women was 14.8% in European Union countries and 10.9% in Portugal [8,9].

By the medical eligibility criteria for contraceptive use outlined by WHO, the use of CHC in the presence of smoking is classified as category 4 (“a condition that represents an unacceptable health risk if the contraceptive method is used”) for women aged ≥35 who smoke ≥15 cigarettes per day and as category 3 (“a condition where the theoretical or proven risks usually outweigh the advantages of using the method”) for women aged ≥35 who smoke <15 cigarettes per day [3]. Therefore, the recommended global clinical decision is to refrain from employing CHC in female smokers aged 35 or older.

We identified the frequent use of CHC among female smokers aged 35 or older as a problem, resulting in an avoidable increase in cardiovascular risk. This quality improvement study aimed to evaluate and improve the quality of hormonal contraception prescription in female smokers aged between 35 and 54 in PC.

## Materials and methods

The present study was conducted in a Portuguese Family Health Unit (FHU), from October 2021 to August 2022, serving around 16 000 patients, including over 2 500 women aged 35 to 54 years old. At the time of the study, the unit comprised nine family doctors, eight nurses, six clinical secretaries and six family medicine residents. This study entails quality improvement of hormonal contraception prescription for female smokers aged 35 years and above in primary care, following the methodology established by the Standards for Quality Improvement Reporting Excellence (SQUIRE 2.0) guidelines and using the Plan-Do-Study-Act (PDSA) approach [10,11].

The study population included all female smokers aged between 35 and 54 years. The upper age limit was set considering that conception after the age of 54 is extremely rare, even among menstruating women, making contraception cessation at 55 years old secure [12].

Using MIM@UF®, a platform of information and monitorization of FHUs, and the International Classification of Primary Care (ICPC)-2, the list of women with the ‘P17 - Tobacco abuse’ code in the active problem list was obtained. Those within the age range of 35 to 54 years were included in this study. A simple random sample of the population was generated through the randomizer.org platform. The sample size was calculated using the Raosoft® Sample Size Calculator (95% confidence interval, 5% precision level, and expected event prevalence of 50%). Data related to age, contraceptive method used, and smoking habits of each participant were collected from clinical records via the SClinico®, an electronic system with all clinical records in primary care. Exclusion criteria were defined as follows: 1) non-users, namely those without consultations since 2019; 2) women who had undergone hysterectomy; 3) ex-smokers, i.e., those with a record of zero cigarettes per day for at least one year; 4) pregnant women. Sampling and data collection occurred in three distinct time points: the baseline evaluation in October 2021, an intermediate evaluation in February 2022, and post-intervention evaluation in August 2022.

In November 2021, after the baseline evaluation, the first intervention took place, involving a clinical session targeted at physicians and nurses. During this session, the obtained results regarding CHC prescription status within the studied population were discussed and a theoretical presentation was performed, reviewing eligibility criteria for CHC usage, according to WHO. The primary objective was to raise awareness among healthcare professionals and to improve hormonal contraception prescription in this population through better counseling and shared decision making. An educational poster addressing this topic was also created for patient awareness and placed in waiting rooms and nursing offices within the FHU. In response to the baseline assessment, and aiming to engage the entire team, a SMART (specific, measurable, achievable, realistic and timely) quality-defining goal was set: to reduce the prevalence of female smokers aged 35 years or above using CHC to ≤5%.

In February 2022, three months after the initial intervention, results from the intermediate evaluation were presented in a new clinical session with physicians and nurses. Based on the results and team discussion, a second intervention was deemed necessary. Each family team committed to identifying their female smoker patients between 35 and 54 years and evaluating the suitability of the contraceptive method used, altering it if needed. This intervention included telephone and in-person consultations. Concurrently, the active updating of smoking habits records and motivation for smoking cessation were reinforced.

The final post-intervention evaluation occurred in August 2022, nine months after the initial intervention. In September 2022, final results were again presented in a clinic meeting with physicians and nurses.

Data were solely collected by the investigators and stored in a Microsoft Excel® database accessible only to the investigative physicians via password. Descriptive statistical analysis was conducted using Microsoft Excel®, calculating the prevalence of smokers using CHC in each assessment to evaluate the attainment of the defined quality goal.

Confidentiality was ensured by the investigators throughout all phases of the process, with the collected information exclusively used for this study. This study was approved by the *Comissão de Ética para a Saúde da Administração Regional de Saúde do Norte* (Health Ethics Committee of the Northern Regional Health Administration) and the Coordinator of the FHU. The study did not disrupt the regular functioning of the FHU nor incurred any cost for it.

## Results

**Figure 1:**
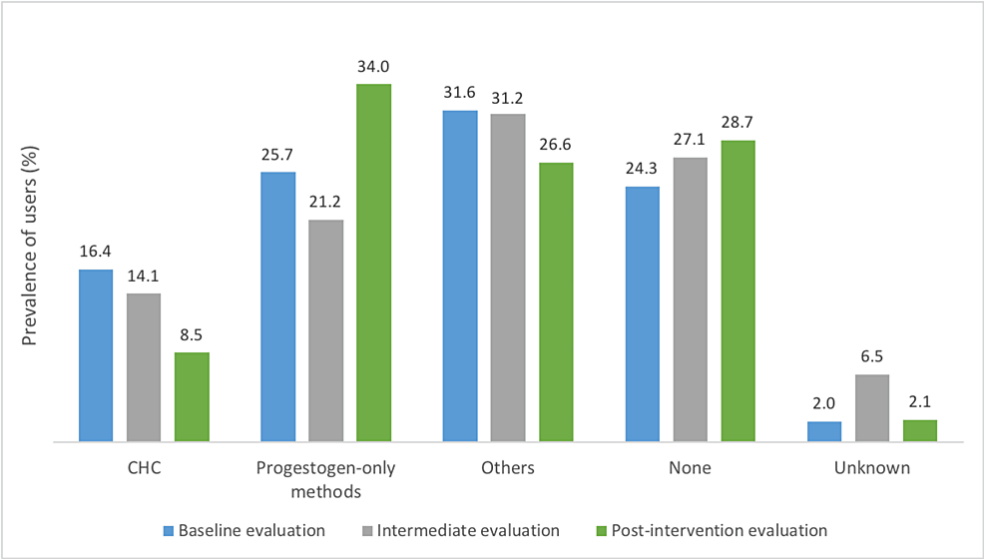
Summarized distribution of contraceptive methods used by women in the studied samples during the baseline evaluation (October 2021), intermediate evaluation (February 2022), and post-intervention evaluation (August 2022) CHC, combined hormonal contraceptives

In the baseline evaluation conducted in October 2021, a total of 527 women aged between 35 and 54 years with the diagnosis “P17 - Tobacco abuse” in the active problem list were identified in the FHU. By calculating the random sample size, a sample of 223 women was obtained, with 71 women excluded. The final sample consisted of 152 participants, with an average age of 44 (SD = 5.6). Among these smoking women, it was found that 16.4% (n = 25) were using CHC (Figure 1). Of these, 12.5% (n = 19) were using combined oral contraceptives, 3.3% (n = 5) were using the vaginal ring, and 0.7% (n = 1) were using the transdermal patch, as described in Table 1. The most commonly used methods were progestogen-only contraception, with a global prevalence of 25.7% (n = 39) (Figure 1). The smoking habits of the sample are described in Table 2, where it was observed that the majority (69.1%; n = 105) smoked less than 15 cigarettes per day.

**Table 1:** Distribution of smoking habits of women in the studied samples during the baseline evaluation (October 2021), intermediate evaluation (February 2022), and post-intervention evaluation (August 2022)

Smoking habits	Number of users at baseline evaluation, n(%)	Number of users at intermediate evaluation, n(%)	Number of users at post-intervention evaluation, n(%)
<15 cigarettes/day	105 (69.1)	122 (71.8)	122 (64.9)
≥15 cigarettes/day	46 (30.3)	43 (25.3)	66 (35.1)
Unknown	1 (0.7)	5 (2.9)	0 (0.0)
Total	152	170	188

**Table 2:** Distribution of contraceptive methods used by women in the studied samples during the baseline evaluation (October 2021), intermediate evaluation (February 2022), and post-intervention evaluation (August 2022)

Contraceptive method	Number of users at baseline evaluation, n(%)	Number of users at intermediate evaluation, n(%)	Number of users at post-intervention evaluation, n(%)
Combined oral contraceptive	19 (12.5)	20 (11.8)	13 (6.9)
Vaginal ring	5 (3.3)	4 (2.4)	3 (1.6)
Transdermal patch	1 (0.7)	0 (0.0)	0 (0.0)
Progestogen-only pill	20 (13.2)	21 (12.4)	33 (17.6)
Contraceptive implant	5 (3.3)	5 (2.9)	10 (5.3)
Progestogen-only injectable	0 (0.0)	1 (0.6)	1 (0.5)
Levonorgestrel intrauterine device	14 (9.2)	9 (5.3)	20 (10.6)
Copper intrauterine device	4 (2.6)	7 (4.1)	5 (2.7)
Barrier methods	25 (16.4)	22 (12.9)	24 (12.8)
Tubal sterilization	16 (10.5)	21 (12.4)	18 (9.6)
Natural method	3 (2.0)	3 (1.8)	3 (1.6)
None	37 (24.3)	46 (27.1)	54 (28.7)
Unknown	3 (2.0)	11 (6.5)	4 (2.1)
Total	152	170	188

At the intermediate evaluation in February 2022, three months after the first intervention, a population of 538 women with the diagnosis “P17 - Tobacco abuse” was obtained, from which, after calculating sample size, a random sample of 225 women was drawn, excluding 55 participants. Among the 170 evaluated women, with an average age of 45 (SD = 5.6), it was found that 14.1% (n = 24) were users of CHC. Combined oral contraceptives remained the most commonly used method (11.8%; n = 20), followed by the vaginal ring (2.4%; n = 4), as described in Table 1. The reduction in the prevalence of CHC usage among smoking women aged 35-54 after three months of the first intervention was 2.3%.

In the final post-intervention evaluation in August 2022, after both interventions, a population of 532 women with the diagnosis “P17 - Tobacco abuse” was obtained. Following the sample size calculation of a random sample of 224 smokers, 36 participants were excluded, resulting in a final sample of 188 women with an average age of 44 (SD = 5.7). The prevalence of CHC usage in the studied sample decreased to 8.5% (n = 16), with 6.9% (n = 13) using combined oral contraceptives and 1.6% (n = 3) using the vaginal ring (Table 1). There was also an increase in progestogen-only contraception usage, with 34.0% (n = 64) of users in the final post-intervention assessment (Figure 1). Regarding smoking habits, the majority (64.9%; n = 122) maintained a consumption of less than 15 cigarettes per day (Table 2).

After nine months of the initial intervention, there was a reduction of 7.9% in the prevalence of CHC usage among smoking women aged 35-54 (Figure 1).

## Discussion

The baseline evaluation revealed a high prevalence of women aged 35-54 who smoke using CHC; it is clear that approximately one in six smoking women in this population had an elevated risk of cardiovascular events, primarily MI [3]. While the defined quality target of reducing the prevalence of smoking in women aged 35 or above using CHC to ≤5% was not fully achieved, the results remained consistent with the goals for prescription improvement outlined in the SQUIRE 2.0 guidelines [10]. Following the initial intervention, CHC usage prevalence slightly decreased from 16.4% to 14.1% within three months. Subsequently, a more structured and proactive family team approach in a second intervention led to a further reduction, with prevalence declining to 8.5% within nine months. Additionally, as one of the safest contraceptive alternatives for smoking women over 35 years old, there was a growing trend in the usage of progestogen-only methods over time.

The outcomes indicating the elevated prevalence of smoking women using oral contraceptives align with findings from other population-focused studies [13,14]. Several hypotheses can be proposed to explain these results, including potential links between oral contraception and increased nicotine addiction [13], women’s lack of awareness regarding the effects of smoking and contraceptive methods [15], family physicians’ limited knowledge about contraceptive counseling [16], or constraints on consultation time [17]. Considering that family physicians wield significant influence in advising about contraceptive method selection and are the primary prescribers of contraceptives worldwide, initiatives such as our study present a crucial opportunity for enhancing the knowledge of family physicians, which can impact patient education and choices.

Although a pertinent topic, we only identified two similar studies worldwide [18,19]. In another quality improvement project targeting adolescents, strategies similar to those employed in our study, such as clinical sessions and the provision of scientifically oriented materials for the physician-nurse team, resulted in a 21.6% improvement in CHC prescriptions at six months and a 45.6% improvement at 12 months, highlighting the potential impact of time-efficient quality improvement projects on patient outcomes [19]. In a broader study, Russell and Wiles demonstrated that a significant proportion of CHC prescriptions in PC did not meet eligibility criteria, primarily due to smoking. With more assertive interventions and PDSA cycles, after an eight-month period, both the safety and quality of contraception prescription were improved, enhancing compliance with medical eligibility criteria [18].

Regarding the strengths of our quality improvement study, we emphasize the collaboration between the medical and nursing teams; it is well-established that this partnership is a chief factor in improving healthcare delivery and patient satisfaction [20,21]. In this study, the intervention involved creating strategies to actively identify patients who would benefit from a contraceptive method change, promoting a commitment from the family team and resulting in a sustained improvement in contraception prescription. Various strategies were used for this verification, including phone consultations or appointments for in-person family planning consultations. Thus, this intervention was pivotal in achieving more significant outcomes, also contributing to the clinical re-evaluation of infrequently visiting patients and promoting healthy lifestyles, particularly smoking cessation. As expected, due to the short period of time that occurred, smoking habits remained similar during this study. Nonetheless, and although the alteration of the contraceptive method is very relevant, the continuous investment in promoting smoking cessation is fundamental to reducing cardiovascular risk, as Europe remains one of the world’s regions with the highest prevalence of smoking, especially among women [22]. Regarding intervention methods, it is recognized that the traditional method of educating physicians on a subject, such as lectures alone, is unlikely to change professional practice, contrary to more interactive interventions [23]. In this study, we not only educated professionals by updating their knowledge about contraception counseling and revising medical eligibility criteria but also implemented this knowledge in practice. This included actively identifying patients with inadequate methods and offering counseling and safer alternatives. In addition to educating and raising awareness among the medical and nursing teams, we also directed efforts toward smoking women through the creation of an educational poster. This poster encouraged them to seek advice from their family doctor or nurse in case of any doubts or concerns. Other strengths included the completion of a PDSA cycle of continuous quality improvement, which encompassed planning, execution, verification, and action. The randomization of samples helped mitigate selection bias. Finally, we followed the SQUIRE protocol to ensure the quality and replicability of the intervention.

With respect to limitations, the small sample size and brief follow-up period hinder drawing statistically significant conclusions and assessing long-term effects. While electronic medical records are very useful in clinical practice [17], clinical inaccuracies, like incorrect registration of smoking habits or contraceptive methods, were an obstacle. Furthermore, the absence of menopause as an exclusion criterion, stemming from the absence of systematic records, presented an additional obstacle, as it led to the inadvertent inclusion of smoking women who do not meet the criteria for any contraceptive method within our study population. Although these limitations were not fully resolvable, this study allowed for patient record updates and reconnection with infrequent FHU users by family physicians and nurses. On the other hand, although women report that their doctor has the greatest influence on their choice of contraceptive method [24], patient resistance was encountered in changing CHC; it is important to consider that such methods are utilized not solely for birth control purposes but also to address issues such as hirsutism, acne, or endometriosis. Yet, this served as an opportunity to reinforce the role of the family doctor and nurse in dissipating myths and advising on the most suitable contraceptive method, according to medical eligibility criteria and every patient’s characteristics. Missed appointments posed a further limitation, impeding counseling and method exchanges in some patients. Also, the studied population typically underutilized healthcare services, which might also have influenced the results. These factors, combined with an ambitious goal, prevented us from fully reaching our target despite some improvements.

In the future, it is intended that the healthcare team reflects on the achieved results and maintains awareness and knowledge to apply contraceptive method eligibility criteria, requiring collaborative efforts of the physician-nurse team and patient empowerment. While conducted in a single FHU, this approach could be replicated in other healthcare units where a high prevalence of female smokers aged over 35 using CHC is observed. To the best of our knowledge, this is the only quality improvement study specifically focused on enhancing contraceptive use among smoking women. The results suggest that interventions that bring together the nursing and medical team, with a focus on improving both medical and patient knowledge about contraception, can have highly significant outcomes in the general population, which is particularly noteworthy when considering the increasing number and high prevalence of female smokers worldwide [25]. Additional research is needed to complement our findings. This involves studies with larger, more representative samples of the general population, longer follow-up periods to evaluate long-term effects, and exploration of different intervention types to compare and comprehend their effectiveness, including interventions involving civil society, public policies, or various patient education and motivation strategies.

## Conclusions

The present study was conducted with a focus on healthcare best practices, aiming to mitigate significant cardiovascular risk factors. A quality improvement study was completed, resulting in the reduction of CHC prescriptions in female smokers aged 35 and above. This was achieved through collaborative efforts of family doctors and nurses as a team, thereby optimizing care delivery to the population. This collaborative endeavor between physicians and nurses is foundational in Family Medicine, as well as the ongoing updating of knowledge to ensure appropriate counseling tailored to each woman’s individual characteristics. In addition to the positive impact on reducing cardiovascular risk within the studied population, the study also facilitated the identification and updating of clinical records, enhancing the positive impact on medical practice as a whole.
